# Stores Licensing Scheme in remote Indigenous communities of the Northern Territory, Australia: a meta-evaluation

**DOI:** 10.1186/s12889-024-19970-0

**Published:** 2024-09-16

**Authors:** Sophie K. Howes, Emma van Burgel, Beau Cubillo, Sarah Connally, Megan Ferguson, Julie Brimblecombe

**Affiliations:** 1 Department of Nutrition, Dietetics, and Food, Be Active Sleep Eat (BASE) Facility, Monash University, Notting Hill, Victoria, Australia; 2 Menzies School of Health Research, Charles Darwin University, Royal Darwin Hospital Campus, Casuarina, NT Australia; 3 School of Public Health, Faculty of Medicine, The University of Queensland, Brisbane, QLD Australia; 4 Curtin School of Population Health, Faculty of Health Sciences, Curtin University, Perth, Australia

**Keywords:** Stores Licensing, Indigenous Peoples, Food security, Indigenous policy, Remote, Australia

## Abstract

A form of food retail regulation called the Stores Licensing Scheme was introduced by the Australian Government in 2007–2022 to ensure food security in remote Indigenous communities of the Northern Territory. We examined evaluations of this Scheme implemented under the *Northern Territory National Emergency Response* and *Stronger Futures Northern Territory* Acts. Grey literature search identified nine primary source evaluations. Reported outcomes were extracted and thematic analysis utilised to determine barriers and enablers. Outcomes included improved availability and quality of groceries, financial structures, and retail practices, albeit not consistently reported. Governance and food cost were perceived barriers. Future policy aimed to improve food security through community stores should consider food cost subsidy, measures to incentivise all stores to improve standards, and improved governance arrangements enabling self-determination for Aboriginal and Torres Strait Islander Store Directors.

**Supplementary Information:**

The online version contains supplementary material available at 10.1186/s12889-024-19970-0.

## Introduction

Indigenous Peoples is a term used to represent diverse social and cultural groups, with an estimated global population of 476 million people across 90 different countries [1]. Indigenous Peoples have ancestral ties and ongoing connections to their lands, waterways, natural resources, and ecosystems to sustain and develop their societies through unique knowledge systems [2, 3]. Colonisation by colonial empires has promoted the subjugation, economic expansion, exploitation and destruction of important ecosystems and natural resources for Indigenous Peoples [4–6]. This has significantly contributed to greater health inequities and socio-economic disadvantage including higher rates of food insecurity, poverty, and an average life expectancy of up to 20 years lower than non-Indigenous peoples [1, 2, 7]. In the Australian context, Indigenous Peoples (herein referred to as Aboriginal and Torres Strait Islander Peoples) are reclaiming connection to their food systems and traditional diets that are linked to good health and wellbeing [8]. However, the impact of colonisation in Australia has restricted access and availability to traditional foods for many Aboriginal and Torres Strait Islander Peoples and historically contributed to food provisioning reliant on first rations followed later by retail stores. There are approximately 74,546 Aboriginal and Torres Strait Islander peoples living in the Northern Territory (NT) of Australia, of which 70% are living on their ancestral lands in areas considered as remote as access to goods and services is highly restricted [9, 10]. Logistical challenges with food provisioning such as limited freight deliveries, seasonal weather disruptions, and extensive food mileage are often experienced in these communities [11].

The retail landscape of remote communities is unique compared to non-remote settings. Aboriginal and Torres Strait Islander Peoples have shaped the rise and development of a remote food retail landscape. Over one third (n = 86) of the approximate 233 stores in Aboriginal and Torres Strait Islander communities in remote Australia are registered as Indigenous Corporations [12]. The onus is often on these (mostly singular) community stores for providing the majority of the community’s food supply [13, 14] alongside household procurement of traditional foods. However, the impact of colonisation as well as climate change has greatly impacted traditional food supply and intake. Therefore, remote community stores significantly influence a community’s ability to achieve food security; defined by members of Aboriginal communities as “when the food of our ancestors is protected and always there for us and our children. It is when we can easily access and afford the right non-traditional food for a collective health and active life. When we are food secure we can provide, share and fulfil our responsibilities, we can choose good food knowing how to make choices and how to prepare and use it” [15]. For many reasons, including the continued effects of colonisation, geographical remoteness impacting on food costs, and lack of employment opportunities within colonial power structures, food insecurity is significantly higher for Aboriginal and Torres Strait Islander Peoples living in remote areas of Australia compared to non-remote areas (31% vs 20%) [16]. For example, during the wet season (November to April) some roads can be inaccessible and subsequently the food supply is disrupted for some communities for several months [13].

In 2007, the Australian Government introduced the Stores Licensing Scheme (herein referred to as Stores Licensing) to regulate remote community stores based on their unique influence on attaining food security in the NT. The first iteration of Stores Licensing occurred under the *Northern Territory National Emergency Response Act 2007* (Cth) (herein referred to as NTER Act) [17]. Licensing enabled stores to participate in one of the NTER Acts; the Income Management scheme, where a proportion of a welfare recipient’s payments was isolated for allowable goods and services, including items in licensed stores [18, 19]. In total, 73 community stores across the NT were subject to the licensing, based on their premises in a prescribed area (defined as an area of Aboriginal land in the Aboriginal Land Rights Act 1976 [20]) and primary operational purpose being the provision of grocery items [17]. Food security parameters were requirements of Stores Licensing, covering the quality, quantity, and range of groceries available, and the promotion and availability of healthy food and drink, as well as financial structure, retail practices and governance of the store [17]. Throughout this review, “Stores Licensing” not only refers to the licensing of stores, but also the proposed food security outcomes of the Acts. Immediately prior to Stores Licensing, the Australian Government established the Commonwealth entity Outback Stores to provide a management service to community stores with a commitment to improve food security in remote Aboriginal and Torres Strait Islander communities. [21].

In 2012, the *Stronger Futures in the Northern Territory Act 2012* (Cth) (herein referred to as the SFNT Act) repealed the NTER Act and renewed Stores Licensing with the aim of “…making sure good food is available in communities” [22]. Stores Licensing under the SFNT Act incurred a number of changes, including more focus on improvement of community store infrastructure and support for underperforming stores. In addition, the scheme was extended across all stores operating in the food security area (defined as the whole area of the NT, excluding urban areas) that the Secretary of the Department deemed to be a key source of groceries for communities, including takeaway and roadhouse stores [22]. Stores licensing under the SFNT Act continued for 10 years, with the sunsetting of the Act in June 2022 [23]. The NT Government has since developed the legislation for the continuation of the scheme, now called the Northern Territory Remote Stores Program.

Both the NTER and SFNT Acts addressed several additional public health priority areas including alcohol management, policing, and housing [17, 23]. Although the primary objective of the NTER and SFNT Acts were not nutrition-related, nutrition-sensitive policy was created with the introduction of measures regarding community food security, thereby having the potential to improve nutrition.

In 2020, a National Agreement on Closing the Gap was made between Aboriginal and Torres Strait Islander Peoples and the Australian Government to collaboratively address the effects of colonisation and inequities [24]. Self-determination is central to this Agreement and is integral to Aboriginal and Torres Strait Islander Peoples having agency over their lives, including participation in policy-making and its implementation. To Close the Gap, it is essential that food security initiatives involving Aboriginal and Torres Strait Islander communities are evaluated to determine their impact. There have been periodic reviews of Store Licensing since its establishment. To our knowledge, however, there is yet to be a comprehensive meta-evaluation of Stores Licensing.

This review aims to examine evaluations of Stores Licensing under the NTER and SFNT Acts. With a focus on the overall outcomes of Stores Licensing and the barriers and enablers to achieving its aims and goals of food security, this review provides the opportunity to inform the next iteration of Stores Licensing under the NT Government (the NT Remote Stores Program) and the National Strategy for Food Security in Remote First Nations Communities [25].

## Methods

A qualitative meta-evaluative approach [26] was utilised to capture all primary research reports related to Stores Licensing under the NTER and SFNT Acts. The Preferred Items for Systematic Reviews and Meta-Analysis guidelines were adhered to. [27] Our methodology was also guided by the methods used in a grey literature systematic review by Godin et al. (2015) [28]. Our meta-evaluation aimed to understand the outcomes and perceived barriers and enablers of Stores Licensing for remote stores in the NT under both the NTER and SFNT Acts.

### Eligibility criteria

Eligibility criteria are outlined in Table 1. Inclusion criteria pertain to the Stores Licensing components of both Acts; NTER and the SFNT [17, 23]. All other schemes related to both Acts were excluded from this review. Despite Income Management being closely interconnected with Stores Licensing, this review focussed on evaluations related specifically to Stores Licensing.

**Table 1:** Inclusion and exclusion criteria for selecting evaluations in this review

	Inclusion	Exclusion
**Type of Publication**	Grey literature Primary evaluation	Formally published in academic sources Public inquiries, bills Secondary research evaluation
**Population**	NT population	Other states and territories in Australia
**Setting**	All of NT	Outside of the NT
**Intervention**	Stronger Futures in the Northern Territory – Stores Licensing scheme NT National Emergency Response Legislation – community Stores Licensing program	Stronger Futures in the Northern Territory – all other schemes (e.g., alcohol restrictions, income management etc.) National Emergency Response Legislation – all other programs
NT; Northern Territory		

### Search resources

Three search strategies were utilised to systematically capture all relevant grey literature: database search, Google search, and targeted website search. Search terms were developed using a Population, Intervention, Comparison, Outcomes (PICO) format in conjunction with referring to known relevant texts on the research topic (Table 2).

**Table 2:** Search terms

	Keywords	POSSIBLE SEARCH TERMS^a^
POPULATION	Australia Northern Territory NT remote stores community stores Aboriginal stores	remote communit* Aboriginal Indigenous store* “community store*”
INTERVENTION	Stronger Futures in the Northern Territory Act 2012 Stronger Futures Act Stronger Futures measures Northern Territory National Emergency Response Northern Territory Intervention Stores licensing Stores Licensing program licensing of community stores licensing regime for community stores community store licensing scheme food security	“Stronger Futures” Intervention NTER Program Regime Scheme store* licens* “food security” MeSH term: Access to Healthy Foods
COMPARISON	Not Applicable	Not Applicable
OUTCOME	review evaluation results performance audit report outcomes	*covered with inclusion/exclusion criteria
FILTERS	2007-now (capturing stores licensing under the Stronger Futures act and the NTER) grey literature (not academically published) Available in English	

^a^truncation/wildcard symbols may vary between search engines/databases

*NT* Northern Territory, *NTER* Northern Territory National Emergency Response, *MeSH* Medical Subject Heading

### Database search

Trove was selected for this review. Trove is the Australian National Library Database that is based on the Australian National Bibliographic Database. The search terms were as follows: “community store*” + licens* + (Emergency OR Stronger). The term ‘community store’ was chosen over other terms representing population as this terminology is used in both Acts [17, 23]. The term represents a store located in the prescribed area whose main purpose is the “provision of grocery items and drinks” [17]. The definition of community store was added to in the SFNT Act to include stores that “consists wholly or partly of selling food, drink and grocery items,” and are located within the food security area [23]. The terms “Emergency” and Stronger” were added to the search strategy to target the type of licensing (Stores Licensing) that the review was focussed on, rather than returning other irrelevant ‘licensing’ results (e.g., driver’s licensing). A search was conducted on the 7th of December 2022 by one author (SH), filtering for texts from 2007 onwards (due to commencement of the first Act) and reports published in English. After filters were applied, categories outside the scope of this review were excluded including images, maps and artefacts, diaries, letters and archives, music, audio and video, and newspaper and gazettes. This method was re-run prior to publication in August 2024 to ensure the dataset reflects the most relevant and up-to-date literature.

### Customised google search

The following search terms were entered into the Google.com search bar on the 7th of December 2022 by one author (SH); [“community store*” + licens* + (Emergency OR Stronger)], using a private search mode to avoid contamination of results. The first ten pages (representing ~ 100 results) were screened, referring to the title and the preview text. Where the title and/or preview text did not provide sufficient context, the executive summary or table of contents was screened to determine eligibility. This method was re-run prior to publication in August 2024 to ensure the dataset reflects the most relevant and up-to-date literature.

### Targeted websites

Targeted websites relevant to the NTER and SFNT Acts were identified by completing a Google search on the name of each Act and Stores Licensing. The search was conducted on the 13th of December 2023 by one author (SH), utilising key words from the inclusion criteria in the search bar of each website. Keywords used for each specific website were documented in a separate Google Sheets spreadsheet for reference. The first ten pages of results were screened, following the same process as the Google search method aforementioned [28].

### Contacting content experts

To ensure that all evaluations had been captured in the above search techniques, contact was made with a key expert in the Stores Licensing area of the Australian Government. An email was sent by the senior author (JB) to the identified expert outlining the purpose of this review, eligibility criteria, and a list of the current documents included in the review, asking if they were aware of any additional documents that may answer the research question. One additional document was identified and screened by two authors (SH, EvB), before being added to the final dataset.

### Study selection

All documents from the three search strategies were uploaded into Covidence (Melbourne, Australia). Screening was conducted in two stages. After removal of duplicates, documents were screened by two authors (SH, EvB). Due to the nature of grey literature, abstracts were not available in most texts for screening. Documents were screened based on their title and executive summary (where applicable), or contents table for potential eligibility. Caution was taken in this approach, and when it was unclear if a source was relevant to the search questions it was included for full text screening. Conflicts between authors were resolved via discussion and consultation with the senior author. Two authors (SH, EvB) conducted full text screening against inclusion criteria, and uncertain reports were discussed with the author team. Pearling references during full text screening was also conducted to ensure a maximised coverage of the data set. For texts that had evaluated multiple elements of the Acts (e.g., income management, alcohol restriction), the section titled ‘store licensing’ or ‘community stores’ or ‘food security’ was reviewed.

### Data extraction

A purpose-designed template table in Google Sheets was developed to capture the Act being evaluated and to extract data on: organisation completing the evaluation, commissioner of the study, study aims, study design, sampling, community engagement, region involved, participant characteristics, strengths, and limitations (as reported), key outcomes and reported future directions. Data extraction was carried out by one author (SH) and questions were taken to the author team who met regularly for further input and clarification.

### Quality assessment

The AACODS (Accuracy, Authority, Coverage, Objectivity, Date, Significance) checklist was utilised for quality assessment of the included evaluations [29]. This tool was selected due to its design specifically for critical appraisal of grey literature. Two authors (SH, EvB) independently appraised three evaluations and resolved discrepancies via discussion. One author (EvB) independently appraised the remainder of evaluations. Data related to the questions in the AACODS checklist were extracted into a separate Google Sheets document for reference.

#### Data synthesis and analysis

Utilising NVivo software [30], a ‘coding reliability’ thematic analysis approach [31] was conducted to identify perceived barriers and enablers to Stores Licensing. First, SH read through one evaluation and identified preliminary codes. Data were coded against the two parent codes (barriers and enablers) and child nodes were inductively generated and matched with the corresponding parent code. This formed an initial codebook, and definitions were refined and discussed with the author team. Next, three texts were independently coded by two authors (SH, SC). After each coded evaluation, authors discussed inconsistencies to ensure adherence to the codebook. Inter-rater reliability was determined using Cohen’s Kappa. Substantial agreement was found with inter-rater correlation of 0.76, 0.76 and 0.79 for the three evaluations respectively [32]. SH coded the remaining texts independently with application of the codebook. The author team were consulted when new codes were generated. Thematic analysis was conducted by SH and themes were discussed with the author team.

An inductive approach was utilised to capture the outcomes of Stores Licensing as reported in the evaluations to allow for consistent interpretation. ‘Key outcomes’ were categorised into five domains based on the aims and objectives of the Acts: quality of groceries, promotion and availability of healthy food and drink, financial structures, retail practices, and governance.

## Results

### Evaluation selection

**Fig. 1:**
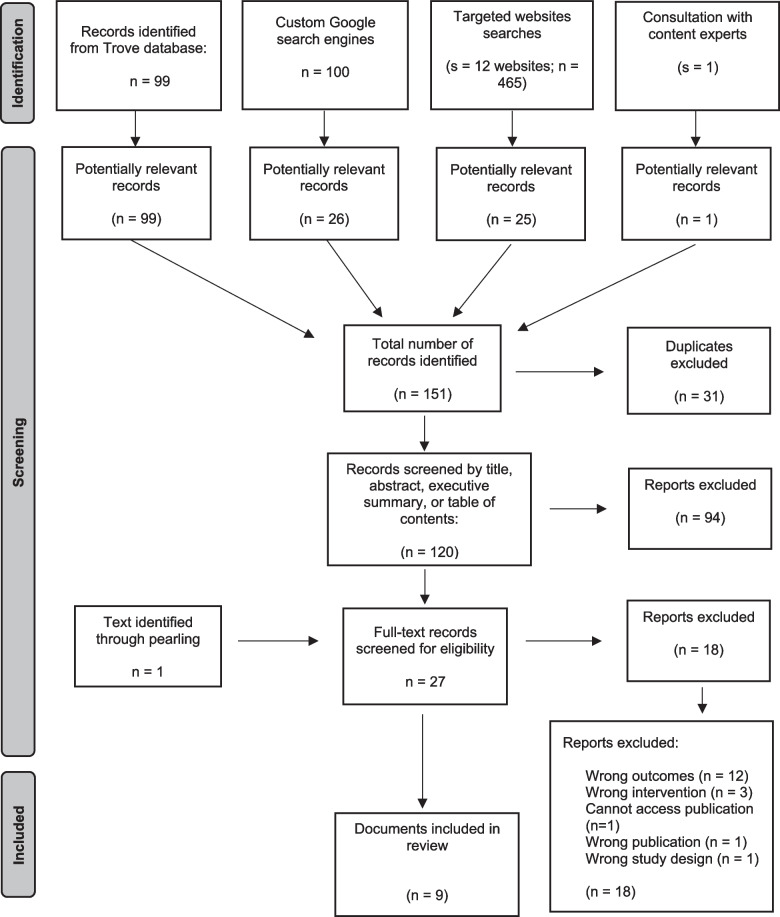
PRISMA flow diagram of meta-evaluation process [27]

After initial screening and removal of duplicates, 120 texts were identified for potential eligibility (Fig. 1). Twenty-seven texts underwent full text screening, including one document identified through pearling. Eighteen results were further excluded, mostly due to reporting on other outcomes related to the Acts (e.g., income management). Nine reports were included in the final dataset.

### Study characteristics

The nine evaluations included in this review [33–41] were published from the year 2008 to 2016 (Table 3). Six reports focussed on the NTER Act [35–38, 40, 41], three reports specifically evaluated the SFNT Act [33, 34, 39], and seven were commissioned by the Australian Government [33–35, 37–40]. Six aimed to evaluate the effectiveness and impact of Stores Licensing, under either the NTER or SFNT Act [17, 23], in the context of food security [34, 35, 37, 39–41]. Three reports focussed on a broader aim of seeking to understand the perspectives and opinions of Aboriginal and Torres Strait Islander communities in relation to the Acts [33, 36, 38]. Most reports [34–37, 39, 41] adopted a multi-methods approach, utilising quantitative data obtained from stores assessments [37], government sources [36], or via survey [35, 41], in addition to collecting qualitative data via interviews [34–37, 39, 41]. (Table 3) An exclusively qualitative methodology was conducted in three reports via extensive community consultation with semi-structured interviews and community meetings [33, 38, 40]. All except two reports [35, 39] involved community consultation. Communities from all regions in the NT were included in six of the reports [33, 36–38, 40, 41], however, specific communities were unable to be determined in the remaining three reports [34, 35, 39] as this was not disclosed or applicable. A range of stakeholders were consulted across the nine evaluations including community store staff, Indigenous and non-Indigenous community members, key stakeholder organisations and Government Business Managers. See Tables 3 and 4 for further detail.

**Table 3:** Overview of study characteristics

**Evaluation Title**		**Methods**	**Sampling**	**Community engagement (Y/N), Region involved, Number of Communities**	**Communities involved**	**Sample size, n**	**Demographics of participants (community members, store managers, board members etc.) age, gender**	**Reported Strengths**	**Reported Limitations**
A Community-based Review of the Northern Territory Emergency Response [41]	**Multi-method** Quantitative - Survey instrument Qualitative - Interviews		Purposive sampling of communities based on: - land tenure system, - governance system, - social and cultural affiliations, - population number, - economic profile, - distance from major service centre	Y	**Communities:** Barunga, Eva Valley, Kalano, Wugularr **Outstations:** Weemol, Emu Springs, Werrenbun, Gorge Camp, Rockhole	**Survey instrument:** - 118 **Interviews:** - Unable to determine	**Survey:** - 69 women, 49 men (21 youth, 85 adults, 13 pensioners) **Interview:** - Aboriginal community members - Non-Aboriginal people employed in communities (managers, nurses, teachers, sports officers) - Townspeople in Katherine (tourist operators, hotel staff, doctors, nurses) - ‘Intensive study’ focus group with five Katherine women (ages 41–75 years)	Nil reported	Risk of bias; Unequal distribution of age and gender from sample; Small sample size for outstation communities
				Katherine East					
				4 communities 5 outstations					
Northern Territory Emergency Response: Perspectives from Six Communities [36]	**Multi-method** Quantitative - Survey - Analysis of Australian Government sources Qualitative - Semi-structured interviews		Purposive sampling based on: - tenure arrangement - size of communities - economic profile, - availability of researchers - date welfare quarantining was due in community - distance from service centre - language and cultural affiliations	Y	Ali Curung, Hermannsburg, Kintore, Papunya, Titjikala, Yuendumu	**Survey instrument:** - 141 **Interviews:** - 51	**Survey:** - Aboriginal residents - Employees from community-based agencies (Council CEO, store manager) - 60% women, 40% men - 77% of participants aged 30–64 years **Interview:** - Employees from community-based agencies (Council CEO, store manager) - Government agencies - GBMs	Nil reported	Age of survey participants skewed towards older adults; Lack of structure to review due to lack of publicly available policy framework; Short time period which limited scope and sample size
				Central					
				6 communities					
Northern Territory Emergency Response Report of the NTER Review Board [40]	**Qualitative** - Community consultation - Interviews with non-government organisations		Purpose sampling based on: - location, - size and type of communities (including outstations and town camps), - degree to which NTER measures had been implemented	Y	Acacia Larrakia, Angurugu, Bagot Town Camp, Barunga, Dagaragu, Galiwinku, Gapuwiyak, Gunbalanya, Hermannsburg, Hidden Valley, Imanpa, Kalkarindji, Lajamanu, Larapinta Valley, Maningrida, Manmoyi, Milikapiti, Mt Nancy Camp, Mutijulu, Nguiu, Ngukurr, Palmerston Indigenous Village, Papunya, Railway Dam, Santa Teresa, Ti Tree, Truckling Yards, Umbakumba, Wadeye, Wugularr, Yuendumu	CD	- Community representatives, community organisations, outstation residents, non-government organisations - Age and gender not specified	Nil reported	Little to no baseline data on the NTER, therefore making it difficult to evaluate the impact of the initiative
				Various					
				31 communities					
Survey of Government Business Managers Relating to the Impact of the Northern Territory Emergency Response [35]	**Multi-method:** - quantitative & qualitative survey		Utilised a census approach, with survey coverage representing 92% of communities	N	N/A	49	GBMs only Age and gender not stated	Nil reported	Nil reported
				N/A					
				GBMs that represent 71 communities					
Report on the Northern Territory Emergency Response Redesign Consultations [38]	**Qualitative** Four-tiered approach: - Tier 1: small group discussions - Tier 2: whole-of-community meetings - Tier 3: Six 2–3-day regional workshops (for community members) - Tier 4: five workshops (with major stakeholder organisations)		Voluntary response sampling and snowball sampling across all four tiers	Y	All 73 NTER communities (at time of evaluation)	Tiers 1&2: - CD Tiers 3 & 4: - 277 people	**Tier 1 & 2:** - Community members (including older traditional men, elders, young women, youth, extended family groups and couples with children) **Tier 3:** - regional leaders **Tier 4:** - representatives of stakeholder organisations (Aboriginal shire councillors and mayors, Indigenous leaders, non-Indigenous community leaders)	Use of Indigenous Engagement Officers and interpreters in consultations	The large scale of arrangements; The diversity of Indigenous languages spoken in the communities (and having a limited number of interpreters); Report not indicative of all the opinions of those affected by the NTER measures
				Widespread					
				All 73 NTER communities & several other Aboriginal communities in the NT					
Evaluation of the Community Stores Licensing Program [37]	**Multi-method** 3 Parts: Quantitative: - Part 1. Data analysis of store assessment data Qualitative: - Part 2: site visits - Part 3: In-depth interviews		Part 1: stratified random sampling based on: - Location - population size of the community - distance to a regional centre - license type; Part 2 & 3: stratified sampling based on: - ownership model - location - community size	Y	(Shires involved): West Arnhem, Barkly, Central Desert, East Arnhem, MacDonnell, Tiwi Islands, Victoria Daly, Roper Gulf) - Specific communities not outlined for privacy	**Analysis of assessment data (part 1):** - 32 stores **Site visits (part 2):** - 4–5 interviews per community (~ 25 total) **In depth interviews (part 3):** - 15	**Stores assessment data (part 1):** - Assessment reports for the Stores Licensing: gathered information on the ‘assessable matters (quality and range of food, promotion of nutrition, retail management, finances, governance) **Site visits (part 2):** - Government staff, store owners/managers, community representatives, committee members - included 2 Indigenous owned stores, 2 OBS-managed stores, and 1 ALPA store **In-depth interviews (part 3):** - a range of stakeholders from key organisations such as FaHCSIA, Outback Stores, ALPA, ORIC, ASIC, Remote Retail Services Gender and age not reported	Nil reported	Assessment report formatting has changed over time—difficult to accurately measure changes over time; Small sample size (of assessment reports); ALPA and OBS also included in samples (who have nutrition policies)—difficult to ascertain the impact of licensing alone
				Widespread					
				(Part 2 & 3): 5 communities					
Stronger Futures in the Northern Territory Report on Consultations [33]	**Qualitative** - Tier 1: small group discussions - Tier 2: whole-of-community meetings Augmented by - Public meetings - Consultations with major stakeholder organisations		Voluntary response sampling	Y	For Tier 1 & 2: Outlined in appendix 2 of report For public meetings: Katherine, Tennant Creek, Darwin, Alice Springs, Nhulunbuy	**Tier 1:** - 378 meetings conducted, CD participants **Tier 2:** - 101 meetings conducted, CD participants **Public meetings:** - ~ 220 **Stakeholder consultation:** - CD	**Tier 1:** - Indigenous community members, community organisations, non-government organisations **Tier 2:** - Open to all community members **Public meetings:** - open to anyone to attend **Stakeholders:** - stakeholders, advocacy organisations (further detail in appendix 3 of report) Gender and age not reported	Nil reported	Data should not be interpreted as being representative of all the views of those consulted in the communities
				CD					
				~ 100 communities for Tier 1 & 2 5 communities for ‘public meetings’ element					
Food Security in Remote Indigenous Communities [34]	**Multi-method:** Quantitative: - analysis of pre-collected data - examined information and documents held by the department Qualitative - interviews - site visits		CD	Y	CD	CD	**Interviews:** - representatives from the Australian Government, representatives from remote store management companies, community store owners and managers, other key stakeholders **Site visits:** - community stakeholders Gender and age not reported	Nil reported	Nil reported
				CD					
				CD					
Review of the Stronger Futures in the Northern Territory Act (2012) [39]	**Multi-method:** Quantitative: - primarily desk-top analysis of existing reports Qualitative: - interviews		CD	N	N/A	N/A	**Interviews:** - Australian and NT Government officials Gender and age not reported	Nil reported	Limited access to quantitative datasets for analysis due to both absence of data and lack of access to existing data
				N/A					
				N/A					

*CD* cannot determine, *N* no, *Y* yes, *N/A* not applicable, *NT* Northern Territory, *GBMs* Government Business Managers, *OBS* Outback Stores, *ALPA*, Arnhem Land Progress Aboriginal Corporation, *ORIC* Office of the Registrar of Indigenous Corporations, *ASIC* Australian Securities and Investments Commission, *FaHCSIA*, Department of Families, Housing, Community Services and Indigenous, *SFNT*, Stronger Futures Northern Territory (Act); NTER; Northern Territory National Emergency Response

**Table 4:** Overview of Included Studies

Reference	Organisation completing review	Year published	Act being evaluated	Commissioner	Reported Purpose of the Evaluation/Aims	Reported Outcomes in Reports of Stores Licensing	Future Directions (as reported)
*A community based review of the Northern Territory Emergency Response* [41]	Institute of Advanced Study for Humanity, University of Newcastle	2008	NTER	N/A	To provide hard data on the impact of the Northern Territory Emergency Response that can inform policy making at community, regional, state, and federal levels	95% of community participants at Barunga report increased good food, 100% at Wugularr and Kalano report no increase; job losses with OBS new management (Barunga); store staff under pressure, decreased store turnover (Kalano); discrepancy in reports about food price (Kalano increased food price, Wugularr unchanged, Barunga even divide)	Address job losses; Consideration for food audit on cost of foods; consider subsidy for healthy foods
*Reviewing the Northern Territory Emergency Response: Perspectives from six communities* [36]	Central Land Council	2008	NTER	N/A	To document the experiences and opinions of Aboriginal people in Central Australia in relation to the NTER	Improved quality and availability of stock; Yuendumu and Ali Curung store boards experienced government pressure (e.g., to comply with the licensing conditions and allow OBS to manage the stores), increased store takings (Hermannsburg); increased food price (Ali Curung, Papunya, Hermannsburg)	Nil reported
*Northern Territory Emergency Response Report of the NTER Review Board* [40]	Australian Government Review Board	2008	NTER	Australian Government (Department of Families, Housing, Community Services and Indigenous Affairs (FaHCSIA))	To consider and comment on the implementation of the measures [of the NTER] and to come to a view about whether they are working or not and, if not, to advise on whether there are better ways of working to address the circumstances of remote communities	Reported ‘impressive’ standards for some stores, others still had ‘high prices, limited range, poor quality and questionable governance arrangements.’	Supports the continuation of the scheme; suggestion for an audit 6-monthly; government to consider subsidy for healthy foods
*Survey of government business managers relating to the impact of the Northern Territory Emergency Response* [35]	TNS Social Research	2008	NTER	Office of Indigenous Policy Coordination Group (OIPC) within the Department of Families, Housing, Community Services and Indigenous Affairs (FaHCSIA)	To develop and test a survey instrument to measure the perceptions of GBMs (Government Business Managers) of how the NTER measures are working on the ground. The aim of the survey is to collect benchmark data that will be used to help assess whether conditions within the communities are improving as a result of the NTER measures	70% and 68% of GBMs reported always having access to frozen vegetables, and frozen meat and poultry respectively; 45% report always having access to fresh fruit and vegetables; 56% report no change to fruit and vegetable quality, 30% report increased quality; 55% report no change to fruit and vegetable availability, 31% reported increased availability. 40% report Stores Licensing has had a positive impact; 42% report no change to food price, 25% report increased prices	Provision of community education about Government’s role in the scheme; consider Outback Stores managing stores and collaborating with community
*Report on the Northern Territory Emergency Response Redesign Consultations* > [38]	Australian Government	2009	NTER	Commonwealth Government	To conduct extensive consultation with Indigenous communities to encourage their input into the NTER legislation	Improved range and quality of food (fruits and vegetables, meat); Improvements in retail practices (store hygiene, management); concerns about governance (future of store committees); no change to prices	Consider more frequent follow-up monitoring of stores; consider price regulation to reduce cost
*Evaluation of the Community Stores Licensing Program: Final report* [37]	Cultural and Indigenous Research Centre Australia (CIRCA)	2011	NTER	Department of Families, Housing, Community Services and Indigenous Affairs (FaHCSIA)	1. Assess outcomes of the licensing regime on store operations, 2. Assess effectiveness of the process, 3. Assess outcomes of the licensing regime on food security. More broadly, the aim of this evaluation was to assess the impact and effectiveness of community store licensing against the assessable matters since the introduction of the NTER legislation on 22 August 2007	Food cost too high; most community members report improvements to quality and range of food, some reported ‘poor’ food quality; improved food promotion (healthy food displays), improved retail practices (stock management, pricing display, storage, shelving, management, hygiene); improved financial structures (removal of book-up, financial transparency requirements), however only ~ 50% of stores meeting gross profit target; No reported improvements to governance (manager and store committee issues)	Suggested improvements for governance: skilled managers, using qualified accountants; suggested more flexibility for assessment process; continue monitoring managers
*Stronger Futures in the Northern Territory Report on Consultations* [33]	O'Brien Rich Research group	2011	SFNT Act	Department of Families, Housing, Community Services and Indigenous Affairs (FaHCSIA)	The purpose of the consultations was to seek the views of people living in the Northern Territory on what to do after the NTER ends in 2012 to tackle the unacceptable level of disadvantage still experienced by Aboriginal people in the Northern Territory	Mixed responses for food quality (good and poor-quality food in stores e.g., fruit, vegetables, meat); mixed responses for food availability (improvements to some stores, others poor availability e.g., during tourist season); governance concerns (calls for including more local people on the store committee, community want more input into the operations of the store); reports of prices being too high (freight costs)	Suggestion for nutrition programs to educate consumers about healthy eating; Government to consider freight subsidies; consider training for store committee members
*Food Security in Remote Indigenous Communities* [34]	Australian National Audit Office (ANAO)	2014	SFNT Act	Department of the Prime Minister and Cabinet	To assess the effectiveness of the implementation of food security initiatives for remote Indigenous communities	Only two-thirds of expected store monitoring visits occurred; prescriptive requirements (from the former legislation) still being imposed; reported confusion in some stores about licensing expectations; ambiguous views on what determines an ‘important source of food, drink, and groceries’ (inconsistent licensing decisions); reported positives to quality and range of stock, and retail management practices (for example the point-of-sales systems); poor data storage from monitoring visits	Ensure monitoring approach is in line with the updated legislation (risk-based); Department to consider ‘strong outcomes focus’ by organising and consolidating performance data more suitably;
*Review of the Stronger Futures in the Northern Territory Act* [39]	KPMG	2016	SFNT Act	Department of the Prime Minister and Cabinet	To evaluate whether the SFNT has contributed to the promotion of greater food security for Aboriginal communities	Reported improved governance and financial management of stores (less reported problems to NT government officials about store hygiene, stock management, finances); improved management (For example, the introduction of criminal history checks); decline in monitoring visits (indicating risk-based approach adherence)	Greater access to consumer information for stores to work collaboratively with community; Government to consider ways of further improving stores (e.g., guidance material provision, nutrition promotion fact sheets, how to improve governance)

*SFNT* Stronger Futures Northern Territory (Act), *NTER* Northern Territory National Emergency Response (Act), *GBMs* Government Business Managers, *OBS* Outback Stores

### Quality assessment

Limited information was disclosed on individual authors for most reports (Table 5). All organisations commissioning or authoring the included reports were deemed reputable and had authority in the field in relation to Stores Licensing. Three of the included reports did not provide reference lists [33, 35, 37].

All reports had a stated aim and methodology. However, four reports [34, 37, 39, 40] did not detail the methodology, making it difficult to ascertain whether methods were appropriate for the aims of the evaluations. Nearly all reports (*n* = 7) were judged as providing an accurate and unbiased interpretation of the data collected. Accuracy and bias were unable to be determined for two reports [34, 39], given the lack of detail in methods, data represented as a summary only, and it was unclear from where conclusions were drawn.

Five reports [33, 36, 38, 40, 41] gave a clear standpoint of the author/organisation, discussing, for example, involvement of Aboriginal people in the organisation completing the evaluation, extensive experience with communities involved in the evaluation, views regarding government responsibility and commitments to building relationships between government and Aboriginal and Torres Strait Islander communities.

Nearly all reports addressed the significance of their evaluations. Two reports [34, 39] did not highlight their evaluation’s representativeness and integrity, where findings of the evaluation represent the wider group, with an unclear methodology adding to the difficulty of determining this.

**Table 5:** AACODS [29] Quality Assessment of included texts

Dimension	Criteria	[41]	[36]	[40]	[35]	[38]	[37]	[33]	[34]	[39]
Authority	Associated with a reputable organisation?	Y	CD	Y	Y	CD	CD	CD	Y	CD
	Professional qualifications or considerable experience?	Y	CD	Y	Y	CD	CD	CD	Y	CD
	Produced/published other work (grey/black) in the field?	Y	CD	Y	CD	CD	CD	CD	Y	CD
	Recognised expert, identified in other sources?	Y	CD	Y	N	CD	CD	CD	N	CD
	Cited by others? (use Google Scholar as a quick check)	Y	CD	Y	N	CD	CD	CD	Y	CD
	Higher degree student under “expert” supervision?	N	CD	N	N	CD	CD	CD	N	CD
	Is the organisation reputable?	Y	Y	Y	N	Y	Y	Y	Y	Y
	Is the organisation an authority in the field?	Y	Y	Y	Y	Y	Y	Y	Y	Y
	Does the item have a detailed reference list or bibliography?	Y	Y	Y	N	Y	N	N	Y	Y
Accuracy	Does the item have a clearly stated aim or brief?	Y	Y	Y	Y	Y	Y	Y	Y	Y
	If so, is this met?	Y	Y	Y	Y	Y	Y	Y	Y	Y
	Does it have a stated methodology?	Y	Y	Y	Y	Y	Y	Y	Y	N
	If so, is it adhered to?	Y	Y	CD	Y	Y	CD	Y	CD	CD
	Has it been peer-reviewed?	N	Y	N	N	N	N	N	N	N
	Has it been edited by a reputable authority?	CD	CD	CD	CD	Y	CD	CD	CD	N
	Supported by authoritative, documented references or credible sources?	Y	Y	Y	N	Y	N	N	Y	Y
	Is it representative of work in the field? If no, is it a valid counterbalance?	Y	Y	Y	Y	Y	Y	Y	Y	Y
	Is the data collection explicit and appropriate for the research?	Y	Y	N	Y	Y	N	Y	N	N
	If item is secondary material, does it refer to the original?	N/A	N/A	N/A	N/A	N/A	N/A	N/A	N/A	N/A
	Is it an accurate and unbiased interpretation or analysis?	Y	Y	Y	CD	Y	Y	Y	CD	CD
Coverage	Are any limits clearly stated?	Y	Y	N	N	Y	Y	Y	N	N
Objectivity	Is the author’s standpoint clear?	Y	Y	Y	N	Y	N	Y	N	N
Date	Does the item have a clearly stated date related to content? If no date is given, but can be easily ascertained, is there a valid reason for its absence?	Y	Y	Y	Y	Y	Y	Y	Y	Y
	Check the bibliography: have key contemporary material been included?	Y	Y	Y	CD	Y	CD	CD	Y	Y
Significance	Is the item meaningful?	Y	Y	Y	Y	Y	Y	Y	Y	Y
	Does it add context?	Y	Y	Y	Y	Y	Y	Y	Y	Y
	Does it enrich or add something unique to the research?	Y	Y	Y	Y	Y	Y	Y	Y	Y
	Does it strengthen or refute a current position?	Y	Y	Y	Y	Y	Y	Y	Y	Y
	Would the research area be lesser without it?	Y	Y	Y	Y	Y	Y	Y	Y	Y
	Is it integral, representative, typical?	Y	Y	Y	Y	Y	Y	Y	CD	CD
	Does it have impact?	Y	Y	Y	Y	Y	Y	Y	Y	Y

*AACODS* Authority Accuracy Coverage Objectivity Date Significance, *CD* cannot determine, *N* no, *N/A* not applicable, *Y* yes

### Reported outcomes

Food security has been defined by several remote Indigenous communities as “when the food of our ancestors is protected and always there for us…It is when we can easily access and afford the right non-traditional food…[and] when…we can provide, share and fulfil our responsibilities, we can choose good food knowing how to make choices and how to prepare and use it” [15]. All of the reported outcomes from this review relate to this definition and represent determinants of food security.

#### Quality of groceries

Based on qualitative data from store operators and community members, three evaluations reported overall improvements in the quality of foods available in community stores since Stores Licensing [34, 36, 38]. Food quality was mostly talked about in the context of broader food groups, including fruit and vegetables, meat, and dairy-based products, rather than specific foods. One study attributed improved food quality to the provision of more funding through Stores Licensing for better store infrastructure, such as refrigerators [37]. Whilst there were positive remarks about Stores Licencing impacting food quality, the Australian National Audit Office (ANAO) audit highlighted that there is unreliable objective performance data to confidently make accurate assessments [34]. In most evaluations, there was discrepancy in responses of whether food quality had improved with the legislation [33, 35, 37, 40, 41]. For example, survey data revealed that 56% of Government Business Managers found no change to the quality of fruits and vegetables, whilst 30% reported increased quality [35]. Community consultation in one evaluation expressed criticism about some stores selling stock beyond its use-by date [33].

#### Promotion and availability of healthy food and drink

Four evaluations reported improvements in the range and promotion of healthy foods in community stores [34, 36–38]. Participants commented on having access to a greater availability of fruits and vegetables in stores since the introduction of Store Licensing. Additionally, store assessment data relating to the Acts’ assessable matters from two time periods indicated a good range of meat, fruit, and vegetables in 90% of stores, which was higher than the previous assessment period [37]. Despite indicating improvements, the assessment criteria changed between assessment periods, and thus cannot be directly compared. Mixed responses were reported in four evaluations regarding food availability [33, 35, 40, 41]. One hundred percent of respondents to a community survey in Wugularr and Kalano expressed no increase in ‘good food’ at their store, whilst 95% at Barunga felt there had been improvements [41]. Interviews with government representatives and community stakeholders offered the perspective that an increased range of healthy foods may be ineffective in the longer term if not accompanied by behavioural nutrition strategies [34]. Most notably, high food cost was reported as a limitation to food accessibility by stakeholders in many communities [33, 35–37, 40, 41].

#### Financial structures

Improvements to financial structures were described in two evaluations [36, 39]. Increased store profits were noted by store staff in the Hermannsburg community stores [36]. However, it is unknown if this is due to increased prices, the introduction of Income Managed funds, or Stores Licensing [36]. Conversely, one report found the Kalano community store to be experiencing financial difficulties, with store turnover described as decreasing from $16,000 to $4,000 per week [41]. The removal of the book-up system under the NTER legislation and financial transparency requirements were positively remarked on by stakeholders, community members and the store committee for one study [37]. However, quantitative data from store assessments in this study found a significant amount of stores were below the recommended gross profit ratio (Tables 3 and 4) [37].

#### Retail practices

Four evaluations reported enhancements to retail practices since Stores Licensing [34, 37–39]. Improvements were outlined in areas such as store cleanliness, environment (including the provision of price labelling and shelving) and management practices. There was consensus that community stores are run similarly to a store in a non-remote area since Stores Licensing, with more structured aisles and shelving for better display of stock. Funding for the point-of-sale system was found to be beneficial, allowing for smoother transactions and processing of Income Managed funds [34]. It was also reported that management practices had improved with the legislation through attracting more well-intentioned managers and driving away those with substandard practices [39]. One report described poor retail practices since Stores Licensing [41]. Interviews with the Barunga store manager highlighted that staff were under greater pressure to keep up with the increased demands [41].

#### Governance

Most evaluations reported ongoing issues related to the governance of community stores [33, 34, 36–38, 40]. Concerns were raised in three reports about the future role of the store committee [33, 37, 38]. It was found that in some stores there were no clearly defined roles between management and the store committee, which in some cases led to dominance of the store manager [33, 37, 38]. Issues were also highlighted in relation to inconsistent assessment and monitoring. The ANAO evaluation found that only two-thirds of the required monitoring visits occurred, highlighting a discordance in the quality of assessment of the Act [34]. Conversely, positive examples of governance were presented in one evaluation, with some stores collaborating with retail consultants and having highly engaged store committees [37].

### Enablers and barriers

**Fig. 2:**
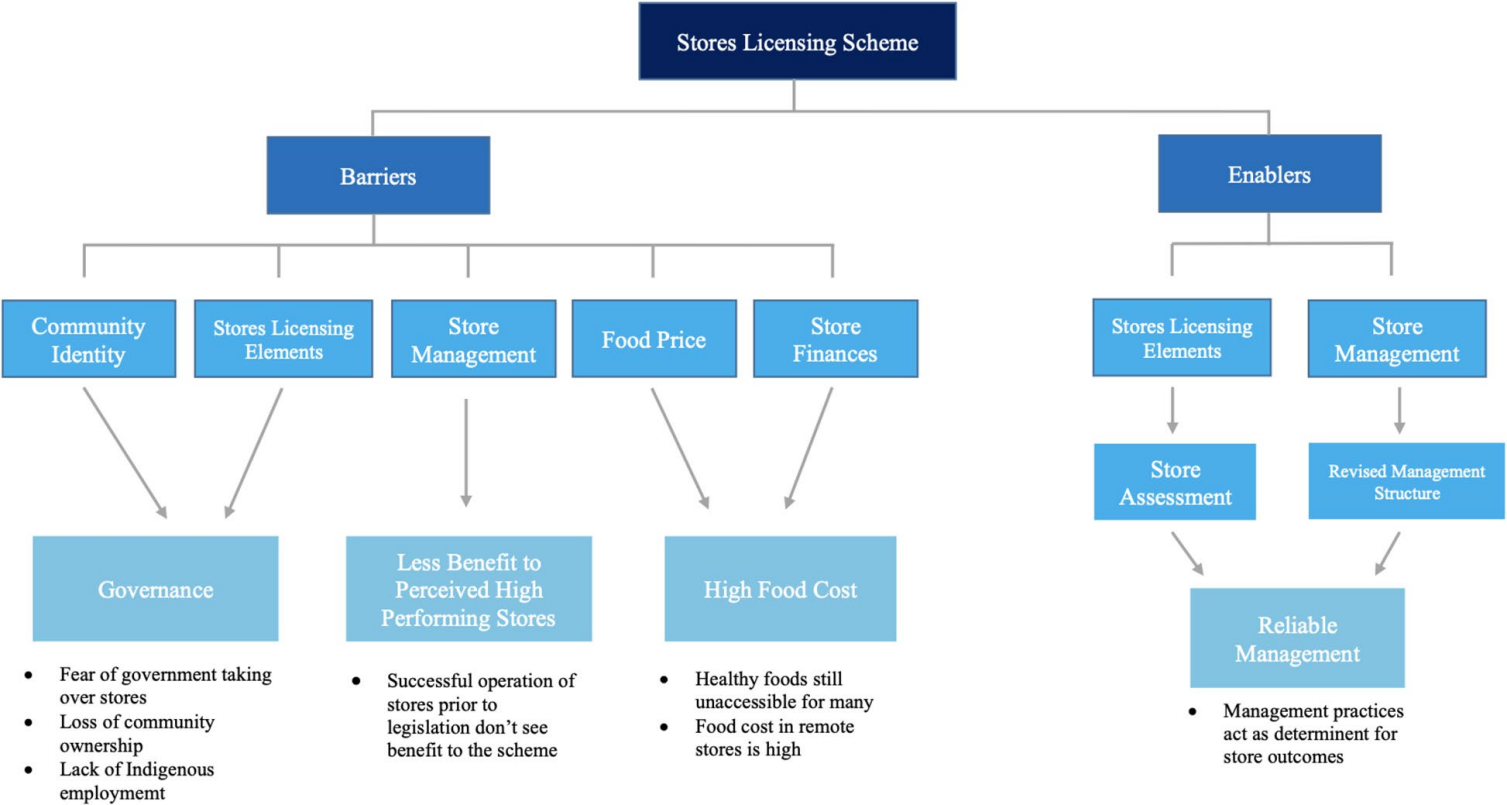
Coding Tree of Perceived Barriers and Enablers to the Stores Licensing Scheme

Based on thematic analysis, four predominant themes were identified in relation to the barriers and enablers of Stores Licensing meeting its aims and goals. The major theme identified as an enabler was reliable management of stores. Three themes were generated as barriers, including governance (such as fear of Government taking over stores), less benefit to perceived high performing stores, and high food cost (Fig. 2).

### Enablers

#### Reliable Management

A key enabler to Stores Licensing was change of store management. It appeared that many of the reported positive outcomes of the initiative (increased stock, improved store layout) were attributed to the introduction of new store managers who facilitated these modifications in line with the legislation requirements. Additionally, appointing Outback Stores to operate selected stores was seen as a positive shift for some community members. Changing to this management structure for selected stores was described as improving the provision of stock and assisted stores in their eligibility to facilitate Income Management. Furthermore, managers that were described as having skilled practices were viewed as operating more efficient and successful community stores.

*“The store is currently in transition to Outback Stores. Already there has been a massive increase in the number of items for sale on the shelves from 210 to 700 items.” *[36]* (Government Business Manager)*

*“The new managers are transparent. Their pricing policy is consistent because there is a certain level of mark-up across the store. The previous manager was selling some stock under-priced.” *[36]* (Government Business Manager)*

It was also highlighted that the assessment process of Stores Licensing further contributed to robust management practices. Under the licensing legislation, the assessable matters, including the character and capabilities of the manager, provide incentive for store managers to adhere to these requirements.

*“Licensing has been a good thing because you can’t get bad shopkeepers who just want to make money and don’t care about the quality.” *[38]* (Community Member)*

### Barriers

#### High food cost

The high cost of food in community stores was a strong theme that was identified. In community consultation, it was often described that prices remained too high even with Stores Licensing, or no beneficial changes to the price of food had been observed. This meant that for many community members, healthy food was still too expensive and unaffordable at community stores. This was a key discussion point across multiple evaluations, suggesting that in many communities making food more affordable may have been an expectation of the initiative in supporting food security, despite it not being an objective of the legislation. Despite the reported improvements to the provision of more variety and stock in stores, for many it was implied that this had no great impact due to the lack of financial accessibility. A consistent recommendation described in the data was a call to the Australian Government to consider subsidising food in community stores.

*“Food is too expensive, especially meat and vegetables. Community members are doing shopping in other places but many people have no vehicle to shop elsewhere.” *[33]* (Community Stakeholder)*

#### Governance

A barrier to Stores Licensing was governance structures that lacked opportunity for input from the community in the decision-making of stores. Common to the data was a sense by community members of reduced community ownership and involvement in the operation of the stores. This was often associated with a new store management structure, or roles and interactions between management and the store committee that were not well defined. This appeared to leave community members feeling disconnected to the store and in some cases fearful of the government taking over the store.

*“We don’t have much say on what happens at the store since the Shire took over…The elders were the ones who signed up and built that store and now they don’t know what’s going on.” *[33] *(Community Stakeholder)*

*“In several of the site visits, community members talked about the governance of the store happening behind closed doors, with limited awareness among community members that they could have an influence on the operation of the store.” *[37] *(Direct quote from text)*

Additionally, a lack of capacity building in relation to Indigenous employment was described. This appears to have further contributed to the disconnect between community members and reduced the support for the Stores Licensing legislation.

*“We want training for Indigenous people to work in the store in finance and stock take. We don’t see our workers doing stock take, only the white workers.”* [33]* (Community Stakeholder)*

#### Less benefit to perceived high performing stores

In certain instances where stores were perceived to be performing well, it was reported that there was less to gain from Stores Licensing, therefore reducing the potential for impact on these stores. This was particularly evident for stores that were managed by Outback Stores and The Arnhem Land Progress Aboriginal Corporation (ALPA). It was described that stores under these management structures already had guiding policies related to promoting food security, similar to those of the Acts. Beyond these organisations, stores that were previously operated by skilled managers also did not see great impact with Stores Licensing.

*“Overall it was felt that for ALPA and Outback Stores, the quality of retail management had not been impacted by stores licensing, because these stores have clear policies and procedures that encourage good retail management processes.” *[37]* (Direct quote from text)*

## Discussion

This review aimed to examine evaluations of Stores Licensing under the NTER and SFNT Acts in remote Aboriginal and Torres Strait Islander communities of the NT. Our focus was to understand the reported outcomes, barriers, and enablers to Stores Licensing in the context of food security. Data suggest that broadly Stores Licensing contributed to improved food security outcomes in community stores, despite some differences in reported outcomes across the evaluations. The barriers identified through this meta-evaluation offer insights to inform areas of improvement for the next iteration under the NT Government and in the National Strategy for Food Security in Remote First Nations Communities [25].

### Policy Implications

#### Food subsidy

Evaluations consistently reported the high cost of food in remote stores as an ongoing barrier to attaining food security. Despite this reporting, Stores Licensing did not have legislative rights to regulate food price in community stores. The ‘assessable matters’ of the NTER and ‘food security matters’ under the SFNT Act focussed on other food security measures as outlined in the Acts [17, 23]. The high cost of food in remote communities has previously been documented, including in the most recent SFNT Sunset Review (2022) and the 2020 parliamentary inquiry into food pricing and food security in remote Indigenous communities [13, 42, 43]. The inquiry highlights the unique retail landscape of remote community stores compared to that of the broader Australian grocery markets. Given their small scale and lack of market competition, community stores are subject to less buying power with wholesalers [43], and can therefore not offer prices similar to urban supermarkets. The high cost of food in remote stores compared to district supermarkets is reinforced in the 2021 Northern Territory Market Basket Survey [44]. According to the report, the average cost of a ‘healthy food basket’ in remote stores was 52% higher than that of district centre supermarkets. Since 2006, the average cost of a healthy food basket in remote stores has progressively increased and been above the projected cost as per the Consumer Price Indexation [44]. This emphasises the persistent upward trajectory of food price in remote community stores, irrespective of inflation, and highlights the price discrepancy between remote and urban settings. There are also logistical challenges that drive food cost in remote settings [11]. Vast distances between freight centres and community stores drive high retail prices. Remote communities are also more exposed to seasonal changes, particularly during the wet season in the NT, which often leads to road disruptions and closures [45]. These factors have a direct impact on not only food price, but also the quality of food. This may explain the discrepancy in reported outcomes for food quality in some community stores, and why improvements in food quality may be beyond the capacity of Stores Licensing alone.

Some community stores including those owned or operated under organisations such as ALPA and Outback Stores have implemented their own food pricing policies to promote healthier food purchases. However, these policies alone have also not been enough to overcome the high cost of food [46–48]. As stated in the Food and Agriculture Organisation’s definition of food security, ‘economic access’ to food is a fundamental pillar [49]. Despite challenges precipitated by the geographic landscape and unique market environment, our findings reinforce the ongoing issue of food price and suggest that disregarding this in the NTER and SFNT Act was perhaps a missed opportunity in addressing one of the key determinants of food security. Overlooking this determinant has potential to further perpetuate the health and wellbeing gap between Aboriginal and Torres Strait Islander Peoples living in remote communities and non-Indigenous peoples. As evident in our results, many stores are calling to government to consider a food subsidy to help ease the challenges of attaining food security in remote settings. The Nutrition North Canada (NNC) is a program implemented by the Canadian government that aims to make healthy food and essential items accessible and affordable for Northern communities whereby many Indigenous populations reside. Part of this program includes a food subsidy for a subset of eligible nutritious foods, with a greater subsidy provided to the most nutritious, perishable foods. The implementation of this initiative in Canada highlights their governments’ prioritisation and commitment to attaining food security, particularly for priority populations within their nation [50]. Similarly, the Queensland Government has recently demonstrated their commitment to addressing the high cost of freight to remote areas and subsequently the impact this has on consumers by introducing a Remote Communities Freight Assistance Scheme. The Scheme aims to reduce freight costs and thus the overall price of eligible essential goods sold in remote communities [51]. In response to the 2020 Parliamentary Food Security Inquiry [43], the Australian Government has supported the recommendation for the development of a food security strategy and have since outlined this as a priority action in the 2023 Closing the Gap Implementation Plan [25, 52]. Government policy must include a food subsidy in remote communities to positively influence food security outcomes in addition to promised investment in improved road and utility infrastructure as indicated in the Australian Government response to the recommendations made by the Parliamentary Inquiry into food pricing and food security in remote Indigenous communities [52]. Continuing to ignore the issue of food affordability for Aboriginal and Torres Strait Islander people in remote Australia will continue to impede attainment of the Close the Gap outcomes.

#### Individualised measures to improve standards

The data suggest that Stores Licensing has improved the standard of management and retail operations of previously perceived low performing stores. Whilst the Stores Licensing legislation outlined requirements in relation to stock management, store layout, and store environment [17, 23], it was implied in the data that much of the success and adherence to these measures was determined by the manager of the store. This is likely attributed to the legislation assessment of the store manager ‘character.’ This finding is supported in an inquiry to the Australian Government where it was described that the attitudes of the store manager play a significant role in determining quality, pricing, and supply of goods stocked in the store [13]. Long before the introduction of Stores Licensing, similar patterns had been documented [53]. One study in the 1990s associated greater nutritional intake in Aboriginal communities with a store manager committed and interested in Aboriginal health [53]. A recent evaluation of a remote store health initiative also found a strong sense of social purpose of store managers and business owners to underpin commitment of a store organisation to health improvement [54]. Thus, monitoring this aspect of community stores can be seen as a strength of the legislation and reduces the likelihood of poor management practices occurring.

However, our meta-evaluation suggests that there was little benefit from Stores Licensing for stores perceived as already operating at a satisfactory level. Given the profound influence that managers can have on the successful operation of a store, those who were operating with integrity and skilled practices prior to the legislation were perceived as already meeting the ‘measurable’ elements of the scheme. This may explain the little or no change to the operations of some community stores observed in our results. In this review, this was particularly relevant for stores operating under Outback Stores. The overarching nutrition purpose guiding Outback Stores operations is “to ensure nutritious, affordable and quality food supply” [55]. With strong foundational policies, it is reasonable to deduce that for Outback Stores the measurable elements for management and retail practices had little incentive or obligation to change the current processes. Whilst this is not a criticism of Stores Licensing, it does suggest that despite the scheme’s objective of improving food security, it is perhaps more pitched at bringing stores up to a baseline standard, rather than widespread improvement. As reflected in the revised SFNT Act, the risk-based approach to assessment (a scoring system where stores deemed as higher risk are monitored more frequently) further illustrates the scheme targeting stores that are less likely up to standard. Future iterations of Stores Licensing may benefit from measures that take a targeted approach based on the unique circumstances of the community store to incentivise all stores and lift the benchmark for higher performing stores.

#### Governance training

Our meta-evaluation revealed an inconsistency in communication and decision making of store operations between management, the store committee and community. Issues related to governance of community stores has previously been documented as an area for improvement [13, 42, 43]. In 2016 a tailored program to encourage capacity building in remote community stores was established by the Office of the Registrar of Indigenous Corporations in conjunction with the National Indigenous Australians Agency and the NT Government [43]. However, this program ceased in 2018 due to a decreased demand, and now only a ‘basic, entry level’ governance training is offered. There has been discussion around the difficulties of ongoing governance training for Store Committee members due to the perceived complexity of governance training and language barriers [43]. The Store Committee are directly responsible for the business operations of the store and also represent their community. Committee members are usually Aboriginal and Torres Strait Islander community members and leaders, speaking a number of languages with a range of differing skill levels and with in-depth insight in to their community [13]. This may suggest that the current governance training structures are insufficient in supporting skill development and indeed may lead to the perceived unequal governance hierarchy described in some stores. Capacity building is defined by the United Nations as “strengthening the skills, instincts, abilities, processes and resources that…communities need to survive, adapt, and thrive in a fast-changing world” [56]. This is an integral component in policy development and commitment to Closing the Gap between Indigenous and non-Indigenous Australians. Therefore, future iterations of Stores Licensing should consider a more rigorous approach to governance training with a tailored, culturally safe approach and one that truly recognises the committee as the decision-making authority for their store. This may act as the catalyst to increasing employment opportunities for Indigenous Peoples and encourage more transparency in the operations of the store.

Australia has been supporting the United Nations Declaration on the Rights of Indigenous People since 2009 that calls for Indigenous Peoples right to have control and input into policies that impact their lives [57]. Whilst the Australian Government has adopted various policies aimed at addressing social determinants impacting many Aboriginal and Torres Strait Islander Peoples, there has been criticism for the absence of self-determination principles and effective participation of Indigenous Peoples within such policies and implementation plans [58]. This is of significance given that most of the evaluations in this study were commissioned by the Australian Government. The quality and approach to an evaluation has potential to be greatly influenced by the commissioning body which is incoherent to both national and international policy recommendations [59]. For Indigenous programs to align with international self-determination principles and national Closing the Gap strategies, it is particularly important that Indigenous leadership is engaged in the commissioning, design, interpretation and development of an evaluation to accurately capture the lived experiences and aspirations of those impacted by the program and ensure culturally sensitive practices that centre Indigenous knowledges [59]. In future evaluations of Stores Licensing, greater transparency is needed regarding commissioning protocols to strengthen the quality, and subsequently the impact that evaluations have on remote communities. A national Indigenous Evaluation Strategy has recently been developed to guide the commissioning of Indigenous evaluations [60]. There is great opportunity for such Indigenous led protocols and evaluations to provide insight for other global Indigenous communities in high income countries to implement strategies and strengthen policies impacting these communities.

### Strengths and limitations

Results should be interpreted with caution with six of our included studies evaluating Stores Licensing under the NTER legislation, and only three under the SFNT Act. Despite the latter being the most recent form of legislation, our findings may be more relatable to the outcomes of the first iteration of Stores Licensing under the NTER Act. This is not a reflection of our sampling strategy per se, but rather the actual number of formal evaluations for this program. In addition, the data used in this review is grey literature and thus were not peer reviewed. Other food security initiatives were also implemented in conjunction to Stores Licensing, including the establishment of Outback Stores. The Aboriginals Benefit Account (now known as the Northern Territory Aboriginal Investment Corporation), Storebiz Program, and Income Management Scheme are programs that may have contributed to the food security outcomes captured in our review. Participants of the evaluations may have inadvertently reported on outcomes related to such programs. Therefore, our findings cannot be entirely attributed to Stores Licensing. Although the author group hold extensive experience and regularly met to answer questions and discuss the methodology, data extraction was carried out by one author for this review. Duplicate quality evaluation and coding was conducted for three studies with the remainder completed by a single author. Despite this, the research team were confident with the analysis after triangulating three duplicate quality evaluations, and any discrepancies that couldn’t be resolved were taken to the senior author. Finally, future meta-evaluations with an Indigenous focus could be strengthened through utilisation of a quality assessment tool that further captures the methodologies of the evaluations in the context of Indigenous values. The quality evaluation tool utilised in this review did not take this into consideration. The Centre of Research Excellence in Aboriginal Chronic Disease Knowledge Translation and Exchange Quality Appraisal Tool may be more suitable for future meta-evaluations [61].

A key strength of this review is the experience of the author group in the context of remote Indigenous communities in the NT and food security. One author was a final year Master of Dietetic Student, and the remainder of the author group hold extensive knowledge and research experience on this topic, ranging from several to over 30 years. One author is Aboriginal and an accepted member of the Larrakia and Wadjigan People groups in the NT with experience applying Indigenous research methodologies. The remaining authors are non-Indigenous but hold vast experience as a research team living and working with remote communities and remote community stores in both Australia and abroad. Another strength is the robust search strategy utilised for this review. We utilised a three-step search process including databases, google, and contacting experts in the field to ensure all formal evaluations were captured for this meta-analysis. Finally, for the qualitative analysis an inductive approach was adopted, which allowed the data to guide our analysis.

## Conclusion

This meta-evaluation provides insight into the outcomes, barriers, and enablers of Stores Licensing under the NTER and SFNT Act. Aboriginal and Torres Strait Islander Peoples face greater food insecurity in remote communities of the NT compared to the wider Australian population. Broadly, this review suggests that Stores Licensing promotes greater food security in remote Aboriginal and Torres Strait Islander communities. However, discrepancy in outcomes amongst evaluations was identified and other government programs and initiatives ran alongside Stores Licensing. The Closing the Gap agreement indicates the necessity for Indigenous-related policy to be designed with cultural sensitivity in such a way that empowers Aboriginal and Torres Strait Islanders to have agency over their lives. Our findings suggest that to support remote Indigenous communities in achieving food security, future iterations of Stores Licensing should take into consideration policy to subsidise healthy food, measures to incentivise all stores to improve their standard, and governance training for Aboriginal and Torres Strait Islander store committees and management enabling self-determination in policy-making for remote stores.

## Supplementary Information

Supplementary Material 1.

## Data Availability

Data is provided within the manuscript or supplementary information files.

## Abbreviations

NTER: Northern Territory National Emergency Response
SFNT: Stronger Futures Northern Territory
NT: Northern Territory
ALPA: The Arnhem Land Progress Aboriginal Corporation
ANAO: The Australian National Audit Office

## Availability of data and materials

All data generated and/or analysed during the current study are available from the corresponding author on reasonable request.
