# Supplementary material for: Stores Licensing Scheme in remote Indigenous communities of the Northern Territory, Australia: a meta-evaluation
Source: BMC Public Health. 2024 Sep 16;24:2520. doi: 10.1186/s12889-024-19970-0 (PMC11406765; doi:10.1186/s12889-024-19970-0)
Supplement: Supplementary file 1 — Supplementary Material 1. [file 12889_2024_19970_MOESM1_ESM.docx]

**Supplementary appendix**

**Supplementary Table 1:** Codebook of parent codes

| **Parent code** | **Description of code** |
| --- | --- |
| **Barrier** | This code refers to structures, mechanisms, and factors that negatively interfere with the Stores Licensing Scheme meeting its goals and aims, in the context of food security.  Example:   - Significant levels of store debt restricted a store from obtaining a license. |
| **Enabler** | This code refers to structures, mechanisms and factors that positively contribute to the Stores Licensing Scheme meeting its goals and aims, in the context of food security.  Example:   - The introduction of store monitoring led to a greater variety of fruits and vegetables available in community stores. |
| **Strength** | This code refers to any *reported* strengths of the methodology or process in which the evaluations were carried out, and how they positively contribute to the study aims.  Example:   - The evaluation captured a range of perspectives through interviewing various members of the community with differing roles and responsibilities. |
| **Limitation** | This code relates to any *reported* weaknesses or limitations identified in the methodology or process in which the evaluations were carried out, and how they negatively interfere with the study aims. Where this is not stated as a clear subheading in evaluations, key words that imply limitations were coded such as:   - “Caution should be taken when interpreting these results due to…” |
| **Policy Context – income management** | This code refers to aspects related to income management that contribute to the outcomes of the Stores Licensing Scheme, in the context of food security. This does not include outcomes of income management that do not relate to food (e.g., financial management). |

**Supplementary Table 2:** Codebook of child codes (barriers)

| **Child Code (for Barriers)** | **Description of code** |
| --- | --- |
| Community identity | This code refers to the beliefs, attitudes and political structures of communities that negatively interfere with the uptake of the government led Stores Licensing initiative.  Example:   - The opinion of some community members is that community stores should be kept separate from the government Intervention. |
| Compliance pressure | This code refers to factors related to the compliance of store licensing that placed pressure on or created conflict for individual community stores. This includes pressure to meet auditing requirements, signing contracts and communication with government representatives.  Example:   - Some communities faced pressure from Government officials to allow Outback Stores to manage the stores. |
| Food price | This code refers to changes to price or costs of items in community stores after implementation of the Stores Licensing Scheme that negatively impact the overarching goals of the initiative.  Example:   - Since introduction of Stores Licensing the prices of food in community stores have increased. |
| Store finances | This code refers to financial difficulties of community stores that may restrict or delay the ability of a store to obtain a license or meet licensing requirements.  Example:   - Significant levels of store debt of a community store are a key concern and does not allow the store to meet the income management criteria. |
| Store management | This code refers to any management practices of community stores that negatively contributed to the Stores Licensing Scheme meeting its aims and outcomes.  Example:   - The previous manager had some strange rules like only allowing one purchase a day, people may have some negative perceptions about the store because of the previous manager. |
| Successful previous store operation | This code refers to robust store practices and functioning prior to the implementation of the Stores Licensing Scheme that are therefore less likely to benefit from the initiative. This includes stores that report not noticing significant changes from the Scheme due to a strong foundational operation.  Example:   - One community reported no improvements to the stock of their store as their store had been working well for some time due to work from the Council. |
| Poor administrative processes | This code refers to poor monitoring or auditing structures within stores that cause difficulty with interpreting or observing the overall effectiveness of a particular measure.  Example:   - Results from the survey make it difficult to ascertain if the increased store takings are from income management or increased store prices.   More about unclear performance measures? |
| Store licensing elements | This code refers to any components or elements related to the design of the Stores Licensing initiative. This includes the auditing, monitoring, and legislative requirements under the Stores Licensing Scheme that contribute to the negative outcomes of the Scheme in the context of food security. This includes monitoring visits, prescriptive requirements for stock and store environment regulations.   - Anything to do with the design of the program   Example:  Officers visit stores to audit the compliance to stores licensing. |
| Environmental Factors | Relates to external factors such as non-controllable environmental events that impact supply, quality and provision of food.  Example:   - Flooding delayed the provision of goods to community stores. |

**Supplementary Table 3:** Codebook of child codes (enablers)

| **Child Code (for Enablers)** | **Description of code** |
| --- | --- |
| Food price | This code refers to changes to price or costs of items in community stores after implementation of the Stores Licensing Scheme that positively contribute to the overarching goals of the initiative.  Example:   - The introduction of Stores Licensing has meant that food prices are now regulated so that store managers cannot keep prices unreasonably high, which has led to a decrease in pricing of food in stores. |
| Store management | This code refers to any management practices of community stores that positively contributed to the Stores Licensing Scheme meeting its aims and outcomes.  Example:   - The store has transitioned to Outback Stores management which has led to a greater variety of fruits and vegetables available in store to purchase. |
| Store licensing elements | This code refers to any components or elements of the Stores Licensing initiative. This includes the auditing, monitoring, and legislative requirements under the Stores Licensing Scheme that contribute to the positive outcomes of the Scheme in the context of food security. This includes monitoring visits, prescriptive requirements for stock and store environment regulations.   - Anything to do with the design of the program   Example:   - Officers visit stores to audit the compliance to stores licensing. |
| Community support | Relates to positive attitudes of community members in relation to the Stores Licensing scheme. |

| Websites |  | Website searched | Search Terms |
| --- | --- | --- | --- |
|  | NT government | https://nt.gov.au/ | "community store*" + licens* + (Emergency OR Stronger) |
|  | Parliament of Australia | <https://www.aph.gov.au/> | "store licensing" "stronger futures" "Northern Territory National Emergency Response" - searched under 'bills and legislation' |
|  | DSS | <https://www.dss.gov.au/> | "community store*" + licens* + (Emergency OR Stronger) |
|  | NIAA | niaa.gov.au | 3 separate searches: "store licensing" + "stronger futures" + "Northern Territory National Emergency Response" |
|  | APO | <https://apo.org.au/> | 4 separate searches: ["community store*" + licens* + (Emergency OR Stronger)] + "store licensing" + "stronger futures" + "Northern Territory National Emergency Response" |
|  | NACCHO | https://www.naccho.org.au/ | "community store*" + licens* + (Emergency OR Stronger) |
|  | HealthInfoNet | <https://healthinfonet.ecu.edu.au/> | "stronger futures" + store licensing + "Northern Territory National Emergency Response" - looking under 'publications' |
|  | Indigenous Justice Clearinghouse | <https://www.indigenousjustice.gov.au/> | 4 separate searches: ["community store*" + licens* + (Emergency OR Stronger)] + "store licensing" + "stronger futures" + "Northern Territory National Emergency Response" |
|  | AMSANT | <https://www.amsant.org.au/> | Searched through 'publications' tab, 'submissions' tab |
|  | ORIC | <https://www.oric.gov.au/> | "store licensing" "stronger futures" "Northern Territory National Emergency Response" |
|  | ANAO | <https://www.anao.gov.au/> | "stronger futures" + store licensing + "Northern Territory National Emergency Response" |
|  | NTCOSS | https://ntcoss.org.au/ | "stronger futures" + store licensing + "Northern Territory National Emergency Response", also looked under 'publications' |

**Supplementary Table 4: Targeted websites and search terms**
